# Hypothesis: Is frequent, commercial jet travel by the general public a risk factor for developing cutaneous melanoma?

**DOI:** 10.12688/f1000research.6962.1

**Published:** 2015-08-24

**Authors:** Harvey Arbesman

**Affiliations:** 1 Department of Epidemiology and Environmental Health, University at Buffalo School of Public Health and Health Professions, Buffalo, NY, 14214-8001, USA; 2 Division of Cancer Prevention and Population Sciences, Roswell Park Cancer Institute, Buffalo, NY, 14263, USA; 3 Department of Dermatology, Clinical and Translational Research Center, University at Buffalo School of Medicine and Biomedical Sciences, Buffalo, NY, 14203, USA

**Keywords:** melanoma, risk factor, epidemiology, jet travel, cosmic radiation

## Abstract

Melanoma incidence has been increasing worldwide over the past 50 years and various risk factors have been identified. Interestingly, multiple studies have shown a multifold increased risk of developing melanoma in jet pilots and airline crew. There has also been a dramatic increase in the availability and frequency of jet travel by the general population during this time period.. Therefore, it is hypothesized that frequent commercial jet travel may represent an additional risk factor for the development of cutaneous melanoma in susceptible individuals of the general public.

## Background

The incidence of cutaneous melanoma in light pigmented individuals worldwide has been steadily increasing over the past 4–5 decades ^1– 4^. Sun exposure, including intermittent exposure, is an important environmental risk factor for melanoma, along with history of sunburn, residence in equatorial latitudes and tanning bed usage ^5– 11^. Genetic risk factors include red hair, family history of melanoma, dysplastic nevi, lightly pigmented skin, tendency to burn, inability to tan, and DNA repair defects ^12^. Phenotypic expressions of gene/environmental interactions are risk factors and include melanocytic nevi (increased total number, multiple atypical [dysplastic], and congenital [particularly large axial lesions with multiple satellites]), ephelides, and personal history of melanoma ^12, 13^. Non-solar occupational risk factors have also been shown to be associated with the development of melanoma ^14^. Since no specific currently recognized risk factor adequately explains the rapidly increasing incidence among the general population, the identification of new risk factors that could play a role in melanoma prevention is needed.

Studies from many countries have shown a significant increase in the risk of melanoma in commercial and military pilots; these range from approximately 2 to10 fold ^15– 20^. An increased risk has also been shown in some studies of cabin crew ^21– 23^. Recently a meta-analysis was published that also showed an increased risk in pilots and cabin crew ^24^.

Though it is unclear why aircrew have an increased risk of melanoma, one proposed explanation regarding their increased risk has focused on the exposure to cosmic ionizing radiation present at cruising altitudes of 30,000 feet or higher ^25– 34^. Currently, commercial jets frequently cruise at an altitude that results in cosmic radiation exposure ^26, 28, 30– 33^. In addition, the cosmic radiation exposure is increased with higher altitude flights and long-haul routes ^25^. Cosmic ionizing radiation contains multiple particles that can damage DNA ^28, 32^. Epidemiologic studies have reported a possible relationship between melanoma and exposure to ionizing radiation in other occupational settings ^35– 37^. A recent systematic review regarding cosmic radiation and cancer assessed the role cosmic radiation plays in the development of cancer as compared with other lifestyle factors ^25^.

Another proposed explanation for the documented increased risk among pilots is their increased exposure to UV light during flight ^24, 38^. Recently an analysis was done and found greater amounts of UVA exposure through the windows of jets ^39^. At this time, it is unclear whether increased UVA exposure, cosmic ionizing radiation, circadian rhythm disturbance and/or other undetermined factors are contributing to this increased risk of melanoma in pilots and cabin crew ^26^.

## Presentation of the hypothesis

It is hypothesized that individuals of the general population who frequently travel by jet plane may also have an increased risk of developing cutaneous melanoma in a similar fashion as pilots and cabin crew. This hypothesized risk factor may play a role in the development of melanoma due to a variety of potentially harmful exposures associated with frequent jet travel that could interact synergistically with other known genetic and environmental risk factors in susceptible individuals. This hypothesis is based on the findings that both pilots and cabin crew, generally healthier than the general public ^18^, and with a prevalence of skin cancer risk factors similar to that of the general public ^21, 40^, have a dramatically higher risk of melanoma.

Since the late 1950s, commercial jets have begun to cruise at an altitude of 30,000 feet or higher. This major change in the flying altitude of air travel is consistent with the temporal nature of the rapid increase in the incidence of melanoma. During the past 50 years, the availability and frequency of jet travel among the general public have increased dramatically. In addition, with the deregulation of the airline industry in the 1970s, and the resulting decrease in fares, a higher percentage of the overall population began to experience jet travel. An ecologic study found an association between accessibility to air travel and the incidence rate of melanoma ^41^.

A widely accepted epidemiologic finding consistent with the proposed hypothesis is that intermittent sun exposure is an independent risk factor for melanoma ^5, 11^. The correlation between melanoma incidence and ‘sun holidays’ ^42– 46^ has been primarily interpreted as secondary to intermittent sun exposure. The proposed hypothesis suggests that the increased risk associated with ‘sun holidays,’ may also be related to jet travel and the resultant exposures associated with jet travel to those vacation destinations. Higher socioeconomic status (SES) is also a risk factor for melanoma development ^42, 47^; and higher SES is associated with increased jet travel and ‘sun holidays,’ ^44, 45, 48^ all findings consistent with the hypothesis.

## Testing the hypothesis

A case-control methodology could be utilized to test this hypothesis. One would assess jet travel histories in melanoma patients and comparable controls, controlling for known risk factors such as age, skin type, genetic host factors and sun exposure history. One would obtain assessments of subjects’ jet travel history in terms of frequency, duration of flights and altitude ^49, 50^. Assessment of travel routes, season of travel and increased sunspot activity during flights should also be undertaken. It would be necessary to disentangle intermittent sun exposure history from jet travel history.

## Implications of the hypothesis

It is hypothesized that frequent commercial jet travel by the general public may increase the risk of developing melanoma due to various harmful exposures associated with frequent jet travel. This melanoma and jet travel hypothesis has potential for reducing melanoma-associated morbidity and mortality and warrants properly designed analytic epidemiologic evaluation to assess the validity of this hypothesis. In addition, if demonstrated to be a risk factor, evaluation of the underlying mechanisms behind this increased risk may lead to the expansion of basic science research of etiologic factors of melanoma and of cancer in general.
