# Supplementary material for: Hypothesis: Is frequent, commercial jet travel by the general public a risk factor for developing cutaneous melanoma?
Source: F1000Res. 2015 Aug 24;4:620. [Version 1] doi: 10.12688/f1000research.6962.1 (PMC4670002; doi:10.12688/f1000research.6962.1)
Supplement: Peer review history

# Peer review: Referee response for version 1

**DOI:** 10.5256/f1000research.7498.r11326

**Published:** 2015-11-30

**Authors:** Gabriella Fabbrocini

**Affiliations:** 1 Department of Dermatology, University of Naples Federico II, Naples, Italy

The hypothesis put forward by the author is interesting, yet hard to prove. it is extremely difficult to separate the weight of individual exposures: the cosmic radiation at high altitudes and that related to sun exposure of the trip. The bibliography is extensive and still valid. Considering the bases from which originates the article, or the increased incidence of melanoma in the last decades and the multifactoriality, it might be interesting to start the case control study that the author sets out to accomplish. It would be necessary to consider some essential factors, including the frequency of air travel, the duration of the flight, altitude, intermittent sun exposure of patients during their trip.

I have read this submission. I believe that I have an appropriate level of expertise to confirm that it is of an acceptable scientific standard.

# Peer review: Referee response for version 1

**DOI:** 10.5256/f1000research.7498.r10379

**Published:** 2015-09-29

**Authors:** Robyn Lucas

**Affiliations:** 1 National Centre for Epidemiology and Population Health, Australian National University, Canberra, Australia

The authors raise the hypothesis that commercial jet travel by the general public may increase the risk of developing cutaneous melanoma through increased exposure to cosmic radiation or UVA radiation. Previous studies have shown an increased risk of melanoma in flight crews and pilots. The hypothesis in relation to UVA seems unlikely, given that most air travellers are well-clothed. The hypothesis in relation to cosmic radiation needs stronger evidence of the measured levels within aircraft. The author proposes a case-control study, but this seems unlikely to provide valid results - the most likely explanation of an increased risk of melanoma in flight crews and pilots is higher sun exposure at the locations that they fly to, especially of the intermittent high dose pattern that confers particular risk of melanoma. However sun exposure is poorly reported (as would be the measure of exposure in a case-control study) while air travel likely to be well-reported (and able to be validated). It will be impossible to clearly separate these "exposures".  There is likely to be a very high correlation between a history of air travel and of sunny holidays in people with melanoma, such that a history of air travel is a proxy for high dose intermittent sun exposure. Findings resulting from such a study are highly likely to be spurious associations. It is an interesting hypothesis - a start would be to measure levels of cosmic radiation inside passenger aircraft, more clearly make the link between cosmic radiation and melanoma, and assess the body sites affected in cabin crew and pilots who develop melanoma.

I have read this submission. I believe that I have an appropriate level of expertise to confirm that it is of an acceptable scientific standard, however I have significant reservations, as outlined above.

# Peer review: Referee response for version 1

**DOI:** 10.5256/f1000research.7498.r10189

**Published:** 2015-09-02

**Authors:** Desiree Ratner

**Affiliations:** 1 Department of Dermatology, Icahn School of Medicine at Mount Sinai, New York, NY, 10029, USA

The hypothesis, “Is frequent, commercial jet travel by the general public a risk factor for developing cutaneous melanoma?”, provides an interesting perspective on possible reasons for the increasing incidence of melanoma worldwide. The author cites multiple studies documenting a significant increase in melanoma risk among commercial and military pilots as well as cabin crew. Possible reasons for this increase include exposure to cosmic ionizing radiation at cruising altitudes of 30,000 feet or higher, particularly on long haul routes, as well as increased exposure to UV light during flight. Given that a significant portion of the world’s population now takes advantage of the increased availability and frequency of jet travel, it is possible that the increased incidence of melanoma may be, in part, due to increased exposure to cosmic rays and/or UV exposure during high altitude jet travel.

The author suggests that a case-control methodology be used to test his hypothesis, by assessing jet travel histories in melanoma patients and comparable controls, controlling for known risk factors such as age, skin type, and exposure history. It would be necessary to look at frequency of air travel, duration of flights, and altitude, and to disentangle patients’ intermittent sun exposure history from their jet travel history. If the hypothesis that frequent commercial jet travel represents an additional risk factor for developing cutaneous melanoma proves to be true, not only will it be necessary to look at the mechanisms behind this increased risk, but it will also be necessary to develop appropriate screening and preventive measures to decrease the morbidity and mortality to the greatest degree possible from air travel associated melanoma.

I have read this submission. I believe that I have an appropriate level of expertise to confirm that it is of an acceptable scientific standard.
